# Visualization of Transepithelial Passage of the Immunogenic 33-Residue Peptide from α-2 Gliadin in Gluten-Sensitive Macaques

**DOI:** 10.1371/journal.pone.0010228

**Published:** 2010-04-19

**Authors:** Kaushiki Mazumdar, Xavier Alvarez, Juan T. Borda, Jason Dufour, Edith Martin, Michael T. Bethune, Chaitan Khosla, Karol Sestak

**Affiliations:** 1 Division of Microbiology, Tulane National Primate Research Center, Covington, Louisiana, United States of America; 2 Division of Comparative Pathology, Tulane National Primate Research Center, Covington, Louisiana, United States of America; 3 Division of Veterinary Medicine, Tulane National Primate Research Center, Covington, Louisiana, United States of America; 4 Department of Chemistry, Stanford University, Stanford, California, United States of America; 5 Department of Biochemistry, Stanford University, Stanford, California, United States of America; 6 Department of Chemical Engineering, Stanford University, Stanford, California, United States of America; 7 Department of Microbiology and Immunology, Tulane University School of Medicine, New Orleans, Louisiana, United States of America; Charité-Universitätsmedizin Berlin, Germany

## Abstract

**Background:**

Based on clinical, histopathological and serological similarities to human celiac disease (CD), we recently established the rhesus macaque model of gluten sensitivity. In this study, we further characterized this condition based on presence of anti-tissue transglutaminase 2 (TG2) antibodies, increased intestinal permeability and transepithelial transport of a proteolytically resistant, immunotoxic, 33-residue peptide from α_2_-gliadin in the distal duodenum of gluten-sensitive macaques.

**Methodology/Principal Findings:**

Six rhesus macaques were selected for study from a pool of 500, including two healthy controls and four gluten-sensitive animals with elevated anti-gliadin or anti-TG2 antibodies as well as history of non-infectious chronic diarrhea. Pediatric endoscope-guided pinch biopsies were collected from each animal's distal duodenum following administration of a gluten-containing diet (GD) and again after remission by gluten-free diet (GFD). Control biopsies always showed normal villous architecture, whereas gluten-sensitive animals on GD exhibited histopathology ranging from mild lymphocytic infiltration to villous atrophy, typical of human CD. Immunofluorescent microscopic analysis of biopsies revealed IgG+ and IgA+ plasma-like cells producing antibodies that colocalized with TG2 in gluten-sensitive macaques only. Following instillation in vivo, the Cy-3-labeled 33-residue gluten peptide colocalized with the brush border protein villin in all animals. In a substantially enteropathic macaque with “leaky” duodenum, the peptide penetrated beneath the epithelium into the lamina propria.

**Conclusions/Significance:**

The rhesus macaque model of gluten sensitivity not only resembles the histopathology of CD but it also may provide a model for studying intestinal permeability in states of epithelial integrity and disrepair.

## Introduction

CD is an immune disorder induced by dietary gluten from wheat, rye and barley, and manifests mainly as inflammation, villous atrophy and crypt hyperplasia in the small intestine [1]. Gastrointestinal digestion of gluten releases proteolytically resistant peptide fragments such as the 33-merLQLQPF (PQPQLPY)_3_PQPQPF from α_2_-gliadin in wheat gluten [2]. These oligopeptides traverse the intestinal epithelium in individuals with CD by not yet completely known mechanisms. Deamidation of these peptides is catalyzed by TG2, a ubiquitous extracellular enzyme in the gut mucosa [3]–[5]. In genetically predisposed individuals, deamidated peptides bind with high affinity to HLA-DQ2 or 8, [6], [7] the class II major histocompatibility complex (MHC) alleles possessed by nearly all celiac patients [8]. Such DQ2/8-gluten complexes trigger a deleterious immune response [9], [10], which in fully developed CD results in villous atrophy and crypt hyperplasia of small intestine [11] as well as nutritional malabsorption and chronic diarrhea [3].

In parallel to T cell-mediated immune responses to gluten, a humoral immune response comprising production of AGA and anti-TG2 antibodies occurs in active CD [12]. Even though villous atrophy and crypt hyperplasia remain the gold standard for diagnosis [13], detection of autoantibodies to TG2 is now considered a highly reliable predictor of CD [14], being 85% sensitive and 97% specific for disease diagnosis [15]. In celiacs, subepithelial IgA deposits were detected along the crypt basal membranes in amounts proportional to dietary gluten intake [16]. In addition, immunofluorescent microscopy revealed subepithelial IgA and TG2 colocalization [16], [17].

A potential susceptibility factor for CD is a compromised intestinal barrier that facilitates enhanced transepithelial transport of immunotoxic gluten peptides such as the 33-mer [18]. The precise cause of such abnormal permeability remains to be elucidated. For instance, a hormone-like molecule, zonulin, has been proposed as a trigger [19], being upregulated by gliadin [20] resulting in downregulation of the tight junction protein zonula occludens-1 (ZO-1), which physically connects epithelial cells and prevents leakage through intercellular space [21], [22]. Zonulin has been identified as prehaptoglobin-2 (pre-HP2), a multifunctional protein [23]. In an alternative hypothesis, increased intestinal permeability is the result of elevated levels of interferon-γ in the celiac mucosa [24], [25].

Animal models are needed to study mechanisms of increased intestinal permeability and its contribution to transepithelial passage of gluten peptides, their processing into potent antigens, and, ultimately, pathogenesis. Ideally, such an animal model would exhibit those histological and immunological features of CD most relevant to gluten peptide transport and processing, including villous atrophy, tight junction disarray and subepithelial accumulation of TG2-specific autoantibodies. We recently reported that the 33-mer is transported intact across the intestinal epithelium in enteropathic gluten-sensitive rhesus macaques [26]. In this study, we show that such animals also feature disease-dependent subepithelial plasma-like B cells, which stain positive for both TG2 and immunoglobulin (IgG or IgA). To visualize the transepithelial uptake of the 33-mer in vivo, we instilled Cy-3-labeled 33-mer in the duodenum of gluten-sensitive and control macaques and traced it by confocal microscopy. Further, to examine the integrity of the intestinal tight junction barrier, we examined the distribution of ZO-1. Finally, the ZO-1 findings were corroborated by quantitative measurements of plasma haptoglobin.

## Results

### Clinical and serological hallmarks of gluten sensitivity in macaques

**Figure 1:**
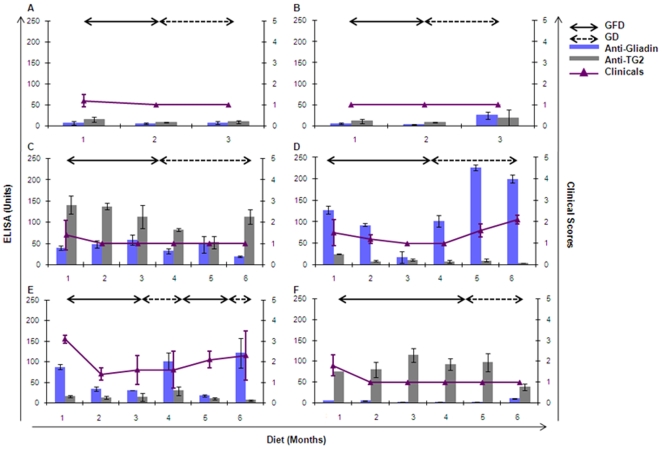
Serological and clinical pre-screening of macaques. Plasma antibody responses to gliadin and TG2 (left axis), and clinical scores (right axis) of negative control (A, B) and gluten-sensitive (C–F) rhesus macaques while on gluten-free diet (GFD) or gluten-containing diet (GD). Cut-off values for anti-gliadin and anti-TG2 antibodies are 50 and 25 ELISA units, respectively.

Based on serological pre-screening of 500 randomly selected colony rhesus macaques, four gluten-sensitive (and two control animals) were enrolled in this study due to presence of high AGA or anti-TG2 antibodies (Figure 1). The gluten-sensitive animals also suffered from non-dehydrating chronic diarrhea of a non-infectious nature. Upon enrollment, all six macaques were placed on GFD to determine if they responded by improvement of clinical scores and decrease in AGA or anti-TG2 antibody levels, as reported recently [27].

Both control macaques A and B remained seronegative for AGA and/or anti-TG2 antibodies while on GD, confirming that dietary changes had no clinical or serological effect on them (Figure 1A, B). Elevated anti-TG2 antibody in macaque C at the time of enrollment showed a decline (p<0.01) after placed on GFD for two months (Figure 1C). When placed back on GD, anti-TG2 antibodies continued to fall for two more months followed by a return to a level comparable to when it was enrolled (Figure 1C). No abnormalities were observed in its AGA profile, regardless of diet (Figure 1C). Initially, its clinical score was 1.5±0.5 (mild diarrhea), but remained clinically normal through most of the study. Macaque D was enrolled due to high level of AGA while on GD (Figure 1D). When administered GFD for two months, AGA decreased (p<0.001) to baseline (Figure 1D), as did clinical scores. When placed on GD for three months, AGA increased (p<0.0001), along with clinical scores (Figure 1D). Anti-TG2 antibodies in this animal were always at baseline levels. In a subsequent cycle of GD and GFD (data not shown), AGA increased again (>200 ELISA units) and clinical scores worsened to 2.0+0.5 on GD, but improved within two weeks on GFD. Macaque E was enrolled with an elevated AGA level. It became negative (p<0.0001) for AGA within one month of GFD (Figure 1E). After being on GD for one month, AGA increased (p<0.001) to a level comparable to that at the time of study enrollment (Figure 1E). A GFD treatment for one month sharply lowered (p<0.01) AGA to a baseline level again, followed by yet another increase after being placed back on GD (Figure 1E). Clinical score decreased from 3 (diarrhea) to 1 (normal) after being placed on GFD for one month, followed by an increase to 2 on one month of GD (Figure 1E). Macaque F exhibited elevated anti-TG2 antibodies at enrollment (Figure 1F). However, these antibodies did not decrease even after three months on GFD (Figure 1F). Clinical scores corresponded to a mild diarrhea, which improved on GFD (Figure 1F).

### Histopathological findings

**Figure 2:**
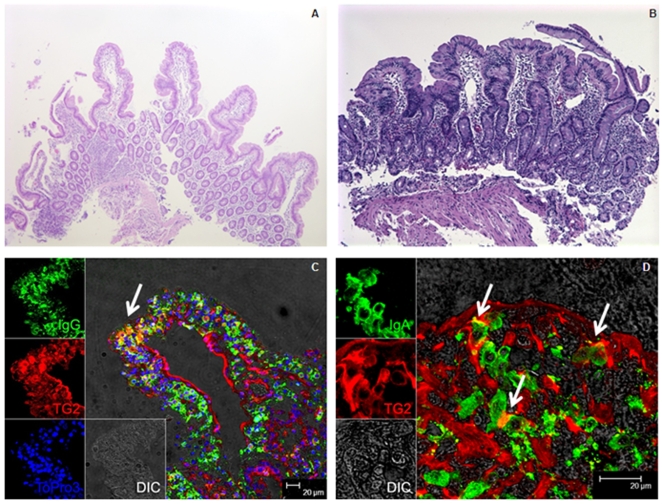
Histopathological and immunofluorescent findings in normal and gluten-sensitive macaque duodenum. An H&E-stained duodenal biopsy from negative control (A) and two gluten-sensitive enteropathic rhesus macaques characterized by shortened, flattened villi and lymphocytosis of lamina propria (B). Biopsies were taken while animals were on GD. Confocal microscopy of duodenal tissue section from a representative animal with gluten-sensitive enteropathy revealed presence of single-positive TG2+ (red), IgG+ /IgA+ (green) cells, as well as TG2+IgG+ (C) and TG2+IgA+ (D) double-positive cells. Double-positive cells are seen as yellow clusters of plasma-like cells due to spectral overlap of red and green (arrows). These cells are suggestive of the source of anti-TG2 antibodies. The TG2 expression was localized mostly in the endothelium while IgG and IgA were localized mostly in the lamina propria. Expression of nuclear DNA is seen in blue (C). Differential interference contrast (DIC) was used for the observation of non-labeled tissues (C, D). Magnification: A & B: 5x; C & D: to a bar scale.

Duodenal biopsies from all four gluten-sensitive macaques were collected at two time points, first while on GD, and second while in clinical and serological remission on GFD. Such biopsies were also collected from the two control animals at corresponding time points. H&E staining of duodenal tissues from gluten-sensitive animals revealed variable levels of tissue inflammation while on GD. All four animals showed intraepithelial lymphocytosis and infiltration of lamina propria with mononuclear cells (not shown). Typical gluten-sensitive enteropathy characterized by shortened and/or flattened villi and crypt hyperplasia was observed in two out of four gluten-sensitive macaques (Animals D & E, Figure 2B). No changes were seen in controls (Figure 2A).

### Double-positive TG2+IgA+ and TG2+IgG+ autoantibody-secreting cells

Duodenal biopsies were processed for confocal imaging [28]. Single positive TG2+, IgG+ and IgA+ cells were readily detectable in all six animals. TG2+ cells were localized mostly in endothelium and lamina propria, and IgG+ and IgA+ cells in lamina propria. Double positive TG2+IgG+ and TG2+IgA+ cells [visualized in yellow due to spectral overlap of green (IgG/IgA) and red (TG2)] were detected as clusters of plasma-like B cells in lamina propria of two out of four (animals D & E) gluten-sensitive animals (Figure 2C, D), but not detected in controls.

### Transepithelial passage of α_2_-gliadin-derived 33-mer peptide

**Figure 3:**
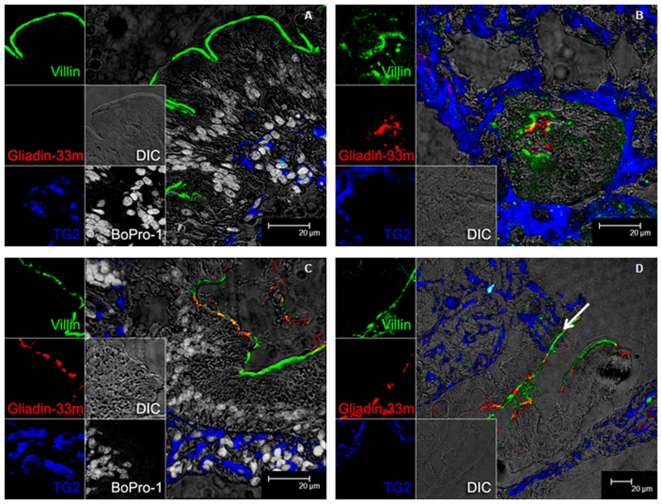
Immunofluorescent detection of 33-mer in macaque duodenum. Duodenal tissue section of the normal control macaque (A and B) on GD. Biopsies were collected at 0 min (A) and 20 min (B) post-instillation with Cy-3-labeled 33-mer, showing the 33-mer in the intestinal lumen and villous layer (B). Panels C and D correspond to gluten-sensitive animal on GD with duodenal biopsies collected at 20 min post-instillation. Panel C shows overlap between the 33-mer and villin markers. Panel D shows incursion of 33-mer into the epithelium plus an area where TG2 left the lamina propria and appeared in epithelium (arrow). In all panels, villin is in green, Cy-3 33-mer in red, and TG2 is in blue. Panels A and C have also nuclear DNA in gray. DIC was used for the observation of non-labeled tissues. Magnification is to a bar scale.

**Figure 4:**
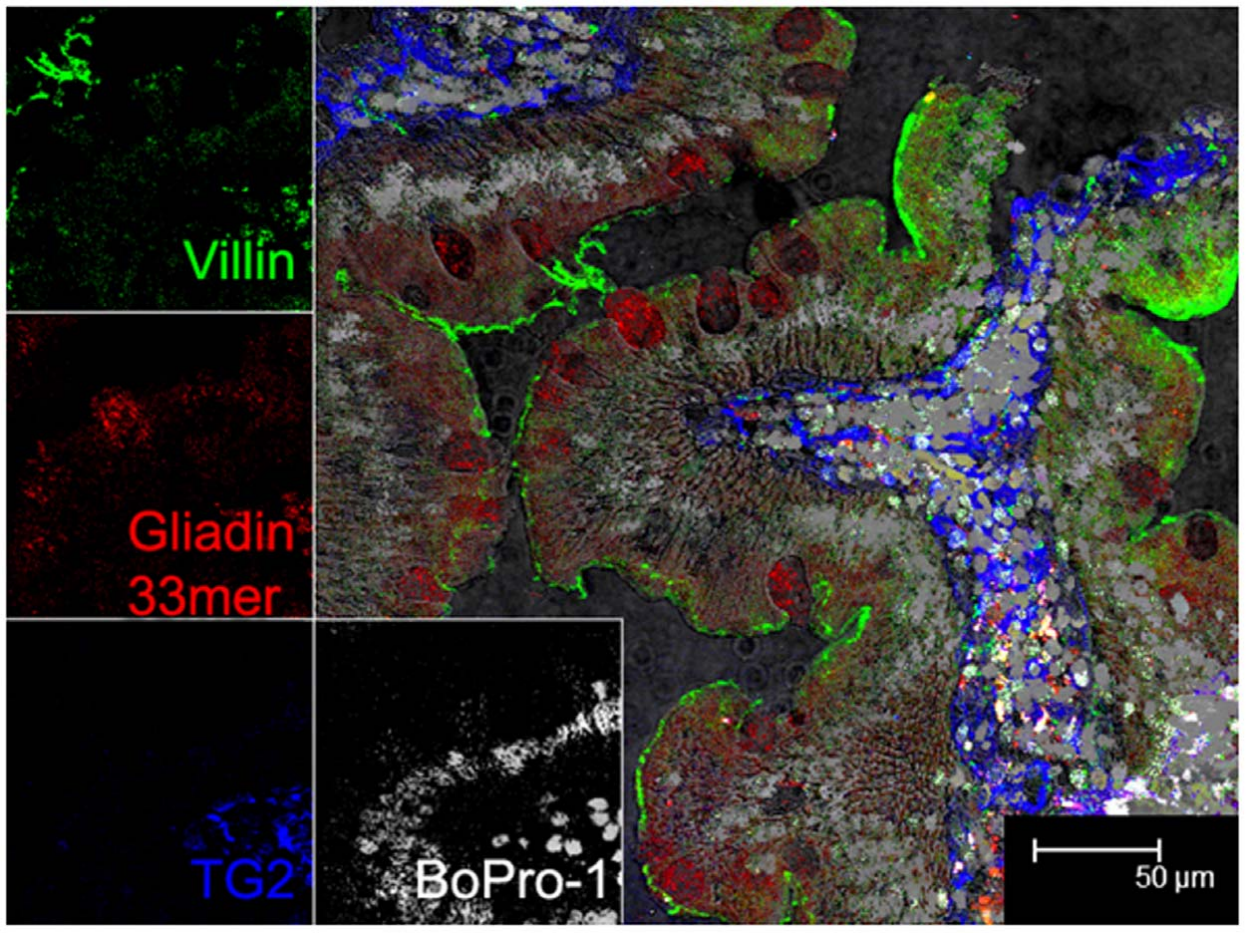
Transepithelial detection of Cy-3-labeled 33-mer in gluten-sensitive enteropathic macaque gut. Duodenal tissue section obtained from the gluten-sensitive enteropathic macaque at 40 min post-instillation with Cy-3-labeled 33-mer. The 33-mer is seen not only inside the epithelium (Goblet cell-like cavities) but also beneath the epithelial layer inside the lamina propria, in close proximity of TG2. Villin is labeled in green, Cy-3 33-mer in red, TG2 in blue, and nuclear DNA in gray. Magnification is to a bar scale.

To study the epithelial entry and transport of the 33-mer gluten peptide, a fluorescent analogue (0.5 g/L Cy-3-labeled 33-mer) was instilled directly in distal duodenum of two healthy controls (animals A & B) and two gluten-sensitive macaques (animals D & E). The latter had significantly elevated plasma AGA levels (p<0.001). All animals were on GD. Pinch biopsies were collected at 0, 20 and 40 min after instillation, immunostained and visualized by confocal microscopy. Cy-3-labeled 33-mer (red) was detected at 20 min post-instillation in association with a villus marker (villin, green) in all macaques (Figure 3). Notably, in gluten-sensitive animal E with pronounced enteropathy, the 33-mer was detected beneath the brush border membrane, inside epithelial cells at 40 min post-instillation (Figure 4). Here, the peptide appeared as red granules inside “Goblet cell-like cavities” (Figure 4). Absence of nuclei in these vacuoles, evidenced by nuclear (BoPro-1) staining and DIC, suggested that these vacuoles could represent gaps left after extrusion of damaged/dying cells from enteropathic gut (Figure 4, Figure S1). Even further penetration of Cy-3 33-mer beneath epithelial layer into lamina propria was seen at 40 min post-instillation in this animal (Figure 4), whereas the peptide was always detected only at the brush border in both controls (not shown).

### Expression and localization of tight junction protein ZO-1

**Figure 5:**
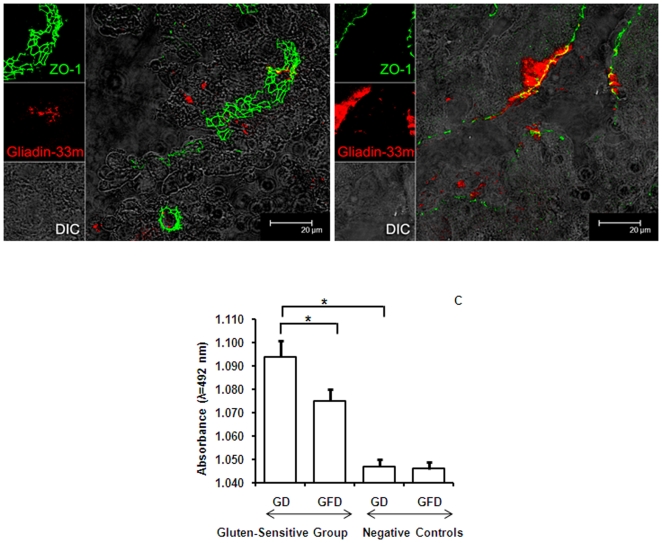
Expression of tight junction proteins in normal and gluten-sensitive macaques. Duodenal tissue sections obtained from normal control (A) and gluten-sensitive enteropathic (B) macaque at 20 min post-instillation with Cy-3-labeled 33-mer. While tight junctions exhibit typical honeycomb staining pattern in control animal, disrupted and weakened pattern is seen in enteropathic animal. Absence of tight junction staining is also seen inside the epithelial gaps (B). Cy-3 33-mer (red) is seen in the epithelial layer. Tight junctions appear green. DIC was used for the observation of non-labeled tissues. Magnification is to a bar scale. Plasma haptoglobin (C) levels in gluten-sensitive and control macaques while on GFD or GD. The averages ± SD reflect the group values as measured by ELISA. Significant (p<0.05) decreases in comparison with gluten-sensitive macaques on GD are indicated by *. Negative sample cut-off  = 1.05 OD units.

To evaluate the integrity of intestinal tight junctions, Cy-3-labeled 33-mer was instilled in the duodenum of gluten-sensitive enteropathic macaques D & E and control animal B while on GD, and biopsies collected as described above were stained with anti-ZO-1 antibody (Table 1). In the control duodenum, ZO-1 exhibited typical continuous honeycomb expression characteristic of intact tight junctions forming a functional epithelial barrier (Figure 5A, Figure S2). In both of the gluten-sensitive enteropathic macaques, ZO-1 expression was markedly reduced to a discontinuous pattern along the luminal side of the epithelial layer (Figure 5B). Importantly, ZO-1 expression was entirely absent from the Cy-3 33-mer-containing gaps observed in the enteropathic macaques, further confirming epithelial barrier loss.

**Table 1:** Antibodies used for immunofluorescent staining of rhesus macaque tissues.

Antibody	Isotype	Working dilution	Manufacturer	Code
Anti-human IgA-FITC *	Goat IgAα	1:100	Sigma-Aldrich	F-5259
Anti-monkey IgG-FITC	Goat IgGγ	1:500	RDI-Fitzgerald	RDI-617102012
Anti-human TG2	Mouse IgG1	1:100	ThermoScientific	MS-300-P1ABX
Anti-human villin	Rabbit IgG	1:3	Cell Signaling Technology	2369
Anti-human ZO-1	Mouse IgG1	1:15	Zymed	33-9100
ToPro-3 (Nuclear DNA)	- **	1:1,000	Invitrogen	T-3605
BoPro-1 (Nuclear DNA)	- **	1:2,000	Invitrogen	B-3583

* Fluorescein isothiocyanate,

** Dye.

### Plasma haptoglobin in gluten-sensitive macaques before and after administration of GFD

Given the altered expression of ZO-1 in small intestine of gluten-sensitive enteropathic macaques, plasma levels of haptoglobin were measured in all six macaques before and after administration of GFD. Compared to healthy controls, gluten-sensitive macaques showed significantly higher (p<0.05) levels of haptoglobin while on GD (Figure 5C). Moreover, the protein level was higher (p<0.05) in gluten-sensitive macaques during the time of AGA or anti-TG2 antibody relapse (GD) than in remission (GFD). The two control macaques showed no change in plasma haptoglobin upon administration of GFD (Figure 5C).

## Discussion

Recently, we described the non-human primate model of gluten sensitivity that shares clinical, histological and serological characteristics with CD [26], [27]. To further investigate the utility of this model, the present study sought to: (i) expand our cohort of gluten-sensitive macaques; (ii) characterize the serological and histological hallmarks of this condition with emphasis on altered intestinal permeability; (iii) visualize transepithelial transport of the immunogenic 33-mer peptide from α_2_-gliadin; and (iv) detect production of TG2-specific autoantibodies in lamina propria.

A total of 500 Indian rhesus macaques were pre-screened for presence of anti-gliadin and/or anti-TG2 antibodies by ELISA. At least four animals were found to be gluten-sensitive based on high levels of the above antibodies and a history of non-infectious chronic diarrhea. This rate (1∶125) is comparable to incidence of CD in humans, ranging between 1∶266 worldwide to 1∶133 in USA [29]. To determine whether there is a similar genetic basis underlying these diseases, it will be important to identify the rhesus analogs of the human MHC II alleles DQ2/8 known to be present in nearly all celiac [8]. Although we recently identified a group of such candidate MAMU II alleles in rhesus monkeys (unpublished data), further corroborative analyses need to be conducted with higher numbers of animals. Our preliminary results suggest that predisposition for gluten sensitivity in macaques might be associated with a broader array of alleles than it is in case of CD and may be independent of MAMU II allele in a subpopulation of animals.

In addition to the four macaques that met our criteria for inclusion in this study, five macaques had increased AGA or anti-TG2 antibodies but no clinical histories of chronic diarrhea. Four of these were asymptomatic, and one presented with extensive skin rash. Both AGA and skin rash disappeared in this animal upon administration of GFD. Asymptomatic or silent forms of CD are common in humans, and we hypothesize that such forms exist also in non-human primates. The observation of extraintestinal manifestation of gluten sensitivity in macaques is in accordance with the highly polymorphic nature of CD [30], [31]. Further studies on this subject are planned.

Three out of four gluten-sensitive animals enrolled (Figure 1C–E) responded well to GFD, as observed by a decrease in AGA or anti-TG2 antibodies. Clinical symptoms were mild but in agreement with serological results (Figure 1). Macaque C had high level of anti-TG2 antibodies, but not AGA (Figure 1C). Macaques D and E responded to dietary changes both serologically and clinically, but during the last phase of GFD treatment of macaque E, clinical scores were still elevated (Figure 1E). It is possible that due to intestinal lesions caused by gluten exposure, macaque E did not recover fully. Macaque F did not respond to gluten withdrawal, as anti-TG2 antibodies remained high (Figure 1F). In humans, GFD-unresponsive cases are referred to as refractory CD [32]. Animal F could represent a similar case of refractory gluten sensitivity. Refractory CD patients often develop cryptic intestinal T-cell lymphomas and phenotypically abnormal intraepithelial lymphocytes, despite benign cytology [33]. Future studies should focus on such features in those gluten-sensitive macaques that do not respond to GFD. Typical gluten-sensitive enteropathy characterized by villous atrophy was observed in macaques D & E (Figure 2B) while variable degrees of lamina propria lymphocytic infiltrations were seen in the other two gluten-sensitive animals but not in controls.

One of the important hallmarks of CD is the presence of circulating autoantibodies against TG2 [15]. TG2 is normally expressed in small intestine, and other isoforms are in skin, brain, liver, kidney, etc [34]. Anti-TG2 antibodies are produced by intestinal mucosa and can be readily detected in intestinal secretions [35], [36]. In active CD, anti-TG2 IgA antibodies target TG2 and form characteristic deposits in small intestinal epithelial membrane [37]. In gluten-sensitive macaques C-F but not in healthy controls, such autoantibody-secreting cells were detected in duodenal lamina propria (Figure 2C, D). Unlike humans, however, no subepithelial IgA deposition was observed.

A major objective of this study was to visualize the transepithelial transport of immunotoxic gliadin peptides across enteropathic (gluten-sensitive) gut. In intestinal lumen, gastrointestinal proteases are the first line of defense against potentially harmful dietary proteins [38]. Clinical studies suggest that an altered intestinal barrier is a predetermining factor for development of CD [18]. Under normal physiological conditions, intestinal epithelium is largely impermeable to gliadin, but in CD patients, gliadin crosses the intestinal barrier and activates the immune system [18]. Several gliadin-specific T-cell epitopes have been identified and reported to cluster in proline-rich regions of the protein [39]. Shan and colleagues (2002) identified the 33-mer (LQLQPFPQPQLPYPQPQLPYPQPQLPYPQPQPF) to be particularly important in that it is highly resistant to proteolytic degradation, contains six partially overlapping copies of three distinct DQ2-restricted T-cell epitopes and is highly stimulatory towards T lymphocytes [2]. Research that addresses how this peptide is transported, how it binds to DQ2 and how it is processed by APCs for presentation to T-cells should facilitate the general understanding of CD [40]. The 33-mer has been detected as a digestive product of ingested gluten in rats and macaques [41], [42]. This peptide can cross the intestinal epithelium in gluten-sensitive enteropathic macaques and appear in blood stream [26]. Notwithstanding, it is yet unclear how gluten peptides are transported intact across mucosal epithelium.

In this study, we visualized transepithelial transport of fluorescently labeled 33-mer directly in duodenum of gluten-sensitive (D & E) macaques. Inspired by the pioneering work of Friis et al., [43] the 33-mer was instilled in distal duodenum, and pinch biopsies taken from the same area up to 40 min post-instillation. While the 33-mer was seen at intestinal epithelium brush border of control and gluten-sensitive macaques (Figure 3B-D), its further penetration beneath epithelium and into lamina propria was seen only in the latter (Figure 4). A recent study involving biopsies derived from celiacs revealed similar pattern [24]. In our study, at 20 and 40 min post-instillation, the 33-mer was detected in close proximity to TG2 in gluten-sensitive enteropathic macaque E, although the two labels did not co-localize. The significance of this finding remains to be elucidated. Further, Cy-3 33-mer was demonstrated inside “Goblet cell-like cavities” in the enteropathic animal. As these cavities lacked nuclear staining, we hypothesize that they were associated with extrusion of epithelial cells (Figure 4, Figure S1). Neither control animal showed such phenomena.

To investigate the molecular basis for elevated transepithelial uptake of 33-mer, enteropathic (D & E) and control (B) macaque duodenal biopsies were stained for tight junctions. The cytoplasmic protein ZO-1 plays a critical role in maintaining tight junction integrity by anchoring occludin to the cytoskeleton via F-actin. In celiacs, ZO-1 expression is downregulated and F-actin is redistributed [21], [22], causing increased intestinal paracellular permeability [21]. The expression and distribution of ZO-1 was evaluated in enteropathic and control macaques on GD. Reduced expression and a discontinuous pattern of ZO-1 was observed at epithelial cell borders in enteropathic macaques, in stark contrast to the control animals, which exhibited a continuous, honeycomb pattern with apical localization of ZO-1 (Figure 5A, B, Figure S2). These findings were corroborated by measurements of plasma haptoglobin. Haptoglobin is associated with several inflammatory and autoimmune diseases including CD [44]. There are three major isoforms (Hp1-1, Hp2-1 and Hp2-2) in humans, predominant in CD patients being Hp2-1, although a higher prevalence of Hp1-1 was found in patients with dermatitis herpetiformis [44]. It is still an open question which isoform/s reflects increased haptoglobin levels measured in gluten-sensitive macaques in this study. It was suggested that in non-human primates, haptoglobin resembles human Hp1-1 [45]. Given the elevated plasma haptoglobin in diseased gluten-sensitive macaques, it may represent a novel hallmark of this condition in primates.

Taken together, our results suggest that intestinal barrier function is compromised in gluten-sensitive enteropathic macaques, similar to celiacs. It is not yet clear how the 33-mer crosses epithelial barrier in small intestine or whether the epithelium plays a role in processing the peptide. Loss of intestinal barrier function and disruption of intercellular tight junctions seem to be pivotal factors facilitating the pathogenesis of CD, and research directed at restoring intestinal barrier function may yield novel treatments. Thus, future studies on intestinal permeability and its modulation using the rhesus macaque model of gluten sensitivity could provide insights toward formulation of such therapies.

## Materials and Methods

### Ethics statement

Approval for all veterinary procedures in this study had been obtained from the Institutional Animal Care and Use Committee (Protocol # 3508), Animal Welfare Assurance A-4499-01. Animals in this project were under the full care of veterinarians of the Tulane National Primate Research Center in accordance with the standards incorporated in the Guide to the Care and Use of Laboratory Animals (NIH) 78–23 (Revised, 1996). All veterinary procedures were performed only with sedated animals.

### Serological pre-screening of rhesus macaques for gluten sensitivity

A total of 500 rhesus macaques (*Macaca mulatta*) of Indian origin, simian retrovirus-free and selected randomly with respect to age and sex, were pre-screened for gluten sensitivity during semi-annual TNPRC colony inventories by testing for AGA and anti-TG2 antibodies by indirect ELISA as previously described [27]. Briefly, a peripheral blood sample (3 ml) was obtained from a femoral vein of each animal while sedated and corresponding plasma was analyzed. The negative cut-off values were determined based on subset of twenty normal healthy animals. The average optical density units +3x SEM were converted to ELISA units. The cut-offs corresponded to ≤50 units for AGA and ≤25 units for anti-TG2 antibody ELISA. Based on the presence of AGA or anti-TG2 antibodies at levels significantly (p<0.001) higher than the negative cut-off, at least four animals out of 500 were determined to be gluten-sensitive. Of these four, two had high AGA and two high anti-TG2 antibodies. All four also had a history of chronic diarrhea of non-infectious nature. Thus, these four gluten-sensitive and two healthy control macaques were enrolled into the study.

### Diets

Two types of diets were used: A) Gluten-containing diet (GD), a commercially available chow routinely used to feed the captive non-human primates (5K63; PMI Nutrition Intl., LLC), containing 20% (by weight) of crude protein including oats and ground wheat; and B) Gluten-free diet (GFD), containing all nutrients at levels identical with GD, except proteins being replaced by gluten-free sources (5A7Q; PMI Nutrition Intl., LLC).

### Clinical evaluation

Clinical scores were recorded daily for all six animals. A blinded clinical scoring (1–6 scale) was followed as described previously [27]. Briefly, average weekly scores were calculated as mean ± standard deviation – based on 7 daily scores.

### Peripheral blood, small intestinal biopsy collection, and diet regimens

Three ml of peripheral (EDTA) blood was collected from a femoral vein of each of the six macaques at the time of study assignment and in bi-weekly intervals thereafter. Plasma was obtained for subsequent testing by ELISA [27]. Pediatric endoscope-guided pinch (pin-head-sized) biopsies (∼10 pieces/site) were collected from distal duodenum of all animals during study assignment. The gluten-sensitive animals were then placed on GFD until AGA or anti-TG2 antibody levels normalized (Figure 1), at which point pinch biopsies were collected again. The two control animals were placed on GFD for a month, after which biopsies were collected again. After collecting the second set of biopsies, gluten-sensitive animals were placed on GD until antibodies increased again. Control animals were placed arbitrarily on GD for one month before undergoing biopsy (Figure 1). Biopsies were fixed in Z-fix (Anatech Ltd., Battle Creek, MI) or 2% paraformaldehyde (USB Corp., Cleveland, OH) in PBS (Gibco-Invitrogen, Carlsbad, CA) for histopathological evaluation and immunofluorescent studies, respectively [28].

### Intraduodenal instillation of α_2_-gliadin-derived, Cy-3-labeled 33-mer

To visualize the α_2_-gliadin 33-mer absorption in an enteropathic gut and further study intestinal permeability in gluten-sensitive macaques, Cy-3-labeled 33-mer (500 µL of 0.5 g/L) was instilled directly into the distal duodenum of two fully sedated gluten-sensitive enteropathic and two control animals, while on GD. Endoscope-guided pinch biopsies (∼5 pieces/site) were collected from the same area at 0, 20 and 40 min post-instillation and fixed in 2% paraformaldehyde for subsequent immunofluorescent staining [28].

### Histopathological examination

Two to three pinch biopsies were fixed in Z-fix and sectioned at 5 µm. Tissue sections were stained with hematoxylin and eosin (H&E), and inspected under light microscope at 5, 10 and 20× magnification in order to detect histopathology typical of CD.

### Immunofluorescent staining and confocal imaging

Two to three pinch biopsies from distal duodenum of each of the four gluten-sensitive and two control macaques were processed [28] for confocal imaging. They were fixed in 2% paraformaldehyde, cryopreserved in 30% sucrose (Sigma, St. Louis, MO) in PBS, and snap-frozen in 25×20×5 mm cryomolds (Sakura Finetek, Inc., Torrance, CA) containing an optimal-cutting-temperature compound (Sakura Finetek). The frozen tissue blocks were cryosectioned (5 µm thick) with a microtome cryostat (Cryostar HM 560 MV; Microm International GmbH, Waldorf, Germany) and subjected to immunofluorescence staining. Briefly, frozen sections were thawed at room temperature for 15 min and permeabilized with PBS containing 0.2% fish skin gelatin (FSG; Sigma) and 0.1% Triton X-100 (Sigma) for 20 min at room temperature. Sections were then washed with PBS-FSG, and blocked with 10% normal goat serum or normal donkey serum (NGS or NDS; Gibco-Invitrogen) in PBS containing 0.2% FSG for 1 hour at room temperature in a humidified slide chamber. Tissue sections were stained with one or combinations of primary antibodies (Table 1) specific for rhesus IgG, IgA and TG2 in order to identify anti-TG2 autoantibodies (IgG or IgA).

For visualizing α_2_-gliadin 33-mer absorption, pinch biopsies collected at 0, 20 and 40 min post-instillation with Cy-3-labeled 33-mer were processed as above, and tissue sections stained for immunofluorescent studies using antibodies (Table 1) specific for villin and TG2, as well as BoPro-1 nuclear DNA stain. To evaluate the intestinal epithelium integrity, expression of a tight junction protein (ZO-1) was examined in the same biopsies by staining with anti-ZO-1 antibodies (Table 1). Each primary antibody staining was followed by secondary isotype-specific antibodies tagged with Alexa Fluor fluorochrome 488 (green), 568 (red) or 633 (blue) (Molecular Probes-Invitrogen) at 1∶1,000 dilution. All antibodies were diluted in 10% NGS or NDS. Sections were washed with PBS–FSG–TX-100 for 5 min, followed by a rinse with PBS-FSG before adding primary or secondary antibodies. Stained tissue sections were mounted with anti-quenching solution (Sigma). Imaging was performed with a TCS SP2 True confocal laser scanning microscope (Leica, Wetzlar, Germany) equipped with three lasers: an argon-krypton laser at 488 nm (green), a krypton laser at 568 nm (red), and a helium-neon laser at 633 nm (blue), that span from the visible to the far-red side of the spectrum. Differential interference contrast (DIC) imaging for observation of non-labeled tissue was also used. All samples were read in a blinded fashion.

### Plasma haptoglobin levels

Plasma haptoglobin levels corresponding to time points of elevated AGA or anti-TG2 antibodies in gluten-sensitive macaques while on GD were compared with those collected while on GFD using indirect ELISA as described earlier [27]. Briefly, 20 µg/ml plasma protein was used for coating, followed by chicken anti-human haptoglobin (Abcam, Cambridge, MA) antibody at 0.4 µg/ml and biotinylated goat anti-chicken (Aves Labs, Inc., Tigard, OR) antibody (1∶500,000) [27]. Plasma from corresponding time points of both control animals were also included. The negative cut-off value, determined as earlier, corresponded to 1.05 optical density units as measured at 492 nm.

### Statistical evaluation

Statistical differences of serological and clinical values between time points corresponding to GD and GFD for each animal were evaluated using Student's t-test. P values lower than 0.05 were considered statistically significant. Prior to enrollment of gluten-sensitive macaques, animals with AGA or anti-TG2 antibodies at levels significantly (p<0.001) higher than the negative cut-off value were selected out of 500 pre-screened macaques.

## Supporting Information

Figure S1 Immunofluorescent detection of 33-mer in Goblet cell-like cavities in gluten-sensitive macaque gut epithelium. Duodenal tissue section obtained from gluten-sensitive animal while on GD. Biopsies were collected at 20 min post-instillation with Cy-3-labeled 33-mer. Confocal microscopy of immunofluorescently labeled tissue sections reveals presence of 33-mer inside the epithelium (arrows). Differential interference contrast (DIC) suggests the absence of nuclei from the Goblet cell-like cavities (A). Further corroboration of such finding was performed with nuclear staining of another tissue section from the same animal (B). Villin is labeled in green, Cy-3 33-mer in red, TG2 in blue, and nuclear DNA in gray (C). DIC was used for the observation of non-labeled tissues. Magnification is to a bar scale.
(0.44 MB TIF)

Figure S2 Typical tight junction morphology in normal macaque duodenum. Duodenal tissue sections obtained from normal control macaque at 20 min post-instillation with Cy-3-labeled 33-mer. Tight junctions exhibit typical honeycomb staining pattern in this animal. Tight junctions appear in green, villin is blue and Cy-3 33-mer (red) is not detected in the epithelial layer. Magnification is to a bar scale.
(0.68 MB TIF)
