# Supplementary figures and images for: Visualization of Transepithelial Passage of the Immunogenic 33-Residue Peptide from α-2 Gliadin in Gluten-Sensitive Macaques
Source: PLoS One. 2010 Apr 19;5(4):e10228. doi: 10.1371/journal.pone.0010228 (PMC2856682; doi:10.1371/journal.pone.0010228)

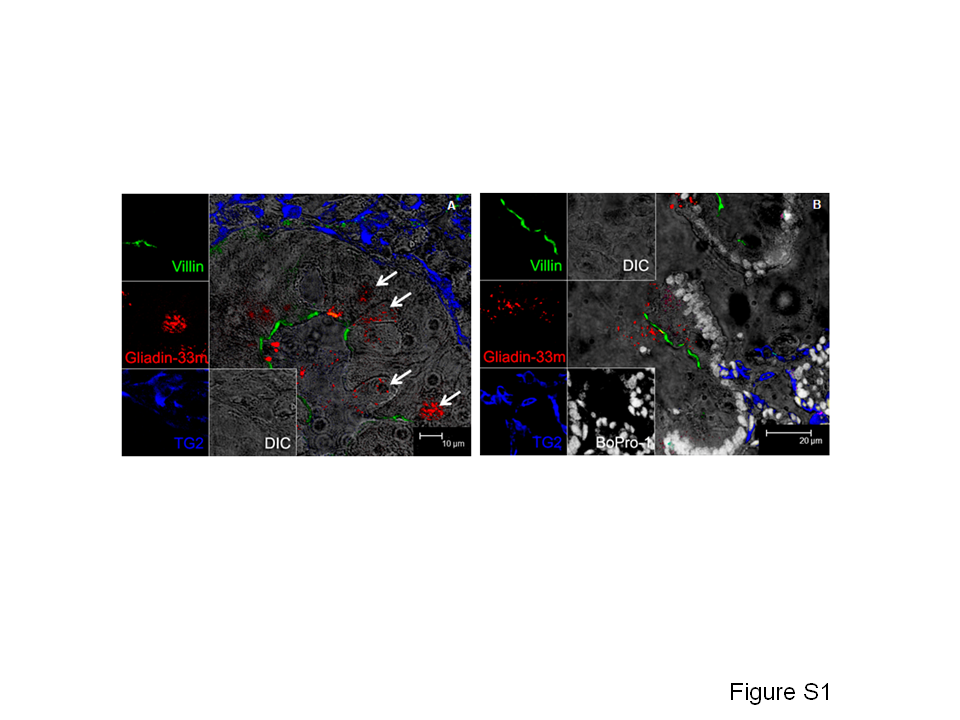

Supplement: Figure S1 — Immunofluorescent detection of 33-mer in Goblet cell-like cavities in gluten-sensitive macaque gut epithelium. Duodenal tissue section obtained from gluten-sensitive animal while on GD. Biopsies were collected at 20 min post-instillation with Cy-3-labeled 33-mer. Confocal microscopy of immunofluorescently labeled tissue sections reveals presence of 33-mer inside the epithelium (arrows). Differential interference contrast (DIC) suggests the absence of nuclei from the Goblet cell-like cavities (A). Further corroboration of such finding was performed with nuclear staining of another tissue section from the same animal (B). Villin is labeled in green, Cy-3 33-mer in red, TG2 in blue, and nuclear DNA in gray (C). DIC was used for the observation of non-labeled tissues. Magnification is to a bar scale. (0.44 MB TIF) [file pone.0010228.s001.tif]

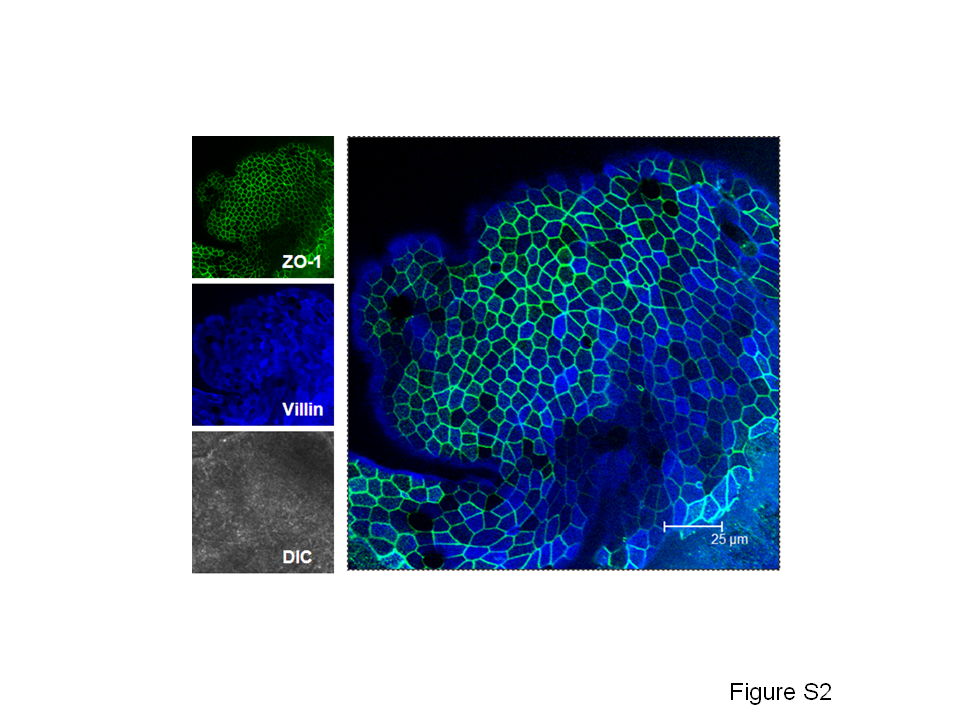

Supplement: Figure S2 — Typical tight junction morphology in normal macaque duodenum. Duodenal tissue sections obtained from normal control macaque at 20 min post-instillation with Cy-3-labeled 33-mer. Tight junctions exhibit typical honeycomb staining pattern in this animal. Tight junctions appear in green, villin is blue and Cy-3 33-mer (red) is not detected in the epithelial layer. Magnification is to a bar scale. (0.68 MB TIF) [file pone.0010228.s002.tif]
